# PCOS stratification for precision diagnostics and treatment

**DOI:** 10.3389/fcell.2024.1358755

**Published:** 2024-02-08

**Authors:** Anagha Joshi

**Affiliations:** Computational Biology Unit, Department of Clinical Science, University of Bergen, Bergen, Norway

**Keywords:** PCOS, stratification, machine learning, women’s health, diagnosis, treatment

## Abstract

Globally, polycystic ovarian syndrome (PCOS) affects approximately 10% of fertile women, leading to great health and economic burden. PCOS is a heterogenous illness that can cause infertility, irregular menstrual cycles, acne, and hirsutism, among other symptoms. The clinical diagnosis is primarily a diagnosis of exclusion if one or more of the three primary symptoms, namely, oligo- or anovulation, hyperandrogenism, and polycystic ovarian morphology, are present. Obesity and PCOS are often coexisting disorders that may be bidirectionally causally related. Phenotypic heterogeneity throughout the reproductive lifespan, such as the overlap of PCOS symptoms with regular fluctuations in a woman’s menstrual cycle and metabolism during the menarche and menopausal transition, further complicates diagnosis. PCOS etiology is mostly unknown and complex, likely due to the fact that it is a group of disorders with overlapping metabolic and reproductive problems. Evidence-based, common, standardized guidelines for PCOS diagnosis and treatment are urgently needed. Genomics and clinical data from populations across diverse ages and ethnicities are urgently needed to build efficient machine learning models for the stratification of PCOS. PCOS subtype-specific strategies for early screening, an accurate diagnosis, and management throughout life will optimize healthcare resources and reduce unnecessary testing. This will pave the way for women to be able to take the best possible care of their own health using the latest clinical expertise combined with their unique needs and preferences.

## 1 Introduction

Polycystic ovarian syndrome (PCOS) is a prevalent heterogeneous disorder affecting 5%–20% of women of reproductive age (Azziz et al., 2016). Even though Stein and Leventhal initially reported the condition in 1935, most PCOS-affected women still go misdiagnosed or undiagnosed. From puberty to old age, PCOS affects women of all ages. In young patients, it primarily manifests itself as menstruation problems, hirsutism, and acne, together with infertility, pregnancy issues, a substantially increasing lifetime risk of type 2 diabetes, cardiovascular disease, and gynecological cancers, such as endometrial cancer. Thus, the clinical consequences of PCOS are observed in a wide range of specialties, including cardiology, geriatrics, dermatology, gynecology, endocrinology, and pediatrics (the Amsterdam ESHRE/ASRM-sponsored 3rd PCOS Consensus Workshop Fauser et al., 2005). An enormous financial and physical burden is caused by PCOS. The estimated cost of diagnosing and treating PCOS is $8 billion, without considering the expense of treating acute reproductive problems, pregnancy difficulties, and long-term metabolic health issues associated with PCOS. Treatment for stroke, type 2 diabetes, and issues related to reproduction, including infertility and hirsutism, is the most expensive component of PCOS care (Riestenberg et al., 2022).

### 1.1 PCOS diagnosis

The first clinical manifestations of PCOS typically appear in adolescence as irregular periods, excessive hair growth, acne, weight gain, and other health issues. When diagnosing PCOS, it is important to rule out any other origins of the phenotype, such as adult-onset congenital adrenal hyperplasia, hyperprolactinemia, and androgen-secreting neoplasms. When combined with other symptoms, hyperandrogenism is recognized as a critical diagnostic factor (Azziz et al., 2006). Globally, there are differences in the methods and criteria used for diagnosis. The National Institutes of Health (NIH) criteria identify PCOS in about 6% of women of reproductive age if they exhibit both hyperandrogenism and oligo/amenorrhea (McCartney and Marshall, 2016). For PCOS diagnosis, the Rotterdam criteria demands two out of the three criteria (Rotterdam ESHRE/ASRM-Sponsored PCOS Consensus Workshop Group, 2004), and the diagnosis can be made with just one polycystic ovary present (Balen et al., 2003). Metabolic risk, specifically insulin resistance, is significantly higher in the NIH group compared to the Rotterdam-defined group. Using the Rotterdam definition raises the prevalence of PCOS in women to up to 20% from 6% using the NIH definition (Broekmans et al., 2006). Laboratory tests measuring free and bioavailable testosterone and androstenedione are used to diagnose hyperandrogenism. Ultrasound scans, blood testing, and clinical symptoms contribute to PCOS diagnosis. These techniques, however, can be expensive, time-consuming, and imprecise. Additionally, a number of PCOS symptoms, such as irregular menstruation and acne, coincide with the typical pubertal stage (Kiconco et al., 2023). The ovaries of women with PCOS are often bigger and contain more follicles. The most popular radiological technique for detecting PCOS is ultrasound; however, the diagnostic cut-offs have been an ongoing debate and have recently been revised. Additionally, multi-follicular ovaries are a common feature around menarche, which limits the utility of ultrasonography to diagnose PCOS for early diagnosis. Currently, the diagnostic cut-offs are dependent on fluctuating laboratory ranges specified by test manufacturers and are based on arbitrary percentiles, frequently from poorly characterized cohorts. This limits the precision of the diagnostic process. A recent study demonstrated that the PCOS diagnostic cut-offs correspond to lower percentiles than conventional PCOS diagnostic criteria (Kiconco et al., 2023). Thus, both physicians and patients are unsatisfied with available diagnosis and treatment options due to diagnostic challenges, delayed and unsatisfactory diagnosis experiences, and less-than-optimal treatment plans (Hoeger et al., 2021). This underscores the pertinent need for precision diagnostics using a combination of novel biomarkers and machine learning methods, with rigorous validation in larger, multi-ethnic, and well-characterized adolescent cohorts. The development of ovarian follicles is regulated by the anti-Müllerian hormone (AMH) during the menstrual cycle. The number of ovarian antral follicles is closely correlated with AMH levels, making a high level of AMH a potential PCOS biomarker (Butt et al., 2022). The broad application of such novel biomarkers is hampered by the absence of an international standard for blood hormone tests, making it challenging to establish consensus criteria (Dumont et al., 2015). Approaches utilizing machine learning have the potential for the identification of PCOS. An ensemble machine learning system was shown by Danaei Mehr and Polat (2022) to be highly sensitive and accurate in PCOS prediction. Using image data from ovarian ultrasound scans, a machine learning algorithm could accurately identify PCOS (Suha and Islam, 2022).

### 1.2 PCOS treatment

Individual circumstances determine the course of treatment for PCOS; similar to diagnosis, there is no single treatment option. As lifestyle changes are shown to improve both the reproductive and metabolic features of PCOS, they are used as the first line of ovulation induction in PCOS patients (Karimzadeh and Javedani, 2010). While rigorous aerobic exercise has been proven to improve body composition, cardiorespiratory fitness, and insulin resistance, no particular diet composition has shown promise in treating the symptoms of PCOS (Cowan et al., 2023). Medical interventions are directed primarily to treat core PCOS symptoms—irregular cycles, hirsutism, and anovulation. Hormonal birth control pills are frequent clinical medications that help regulate menstrual periods and lower testosterone levels. Metformin is advised for obese PCOS women to enhance insulin sensitivity and reduce insulin and blood glucose levels. Studies on the effects of inositol supplementation on metabolic profiles and reduction in hyperandrogenism have demonstrated promise (Cowan et al., 2023). Other pharmaceutical drugs, including ovulatory stimulants like letrozole or clomiphene citrate, are also applied; they induce ovulation by blocking estrogen activity and stimulating the pituitary gland to produce more luteinizing hormone (LH) and follicle-stimulating hormone (FSH), which, in turn, triggers ovulation. When other therapies do not work or are not effective, more intrusive procedures such as bariatric surgery or laparoscopic ovarian drilling are used, which involve destroying a portion of the tissue that produces androgens in an effort to restore ovulation (Hoeger et al., 2021).

## 2 PCOS etiology

All diseases originate as a result of the interaction between genetic and environmental factors. A strong genetic predisposition to PCOS is demonstrated by the twin studies (Vink et al., 2006). Although PCOS pathophysiology is still unclear, genomic variants in genes involved in the regulation of androgen biosynthesis and function are thought to play a role in PCOS pathogenesis (Escobar-Morreale et al., 2005). It is interesting to note that common genetic architecture was noted across different diagnostic criteria, including self-reported diagnosis, by a genome-wide meta-analysis. Numerous metabolic features and PCOS are consistently linked to a high degree of shared genetics (Day et al., 2018). The main environmental factors associated with PCOS are environmental toxins, diet, and nutrition, as well as low socio-economic status (Merkin et al., 2016). It is important to note that the associations based on epidemiological studies, are not substantiated by causal molecular mechanisms.

At first, PCOS was primarily considered an ovarian disorder. It is now believed to be caused by the malfunctioning of the hypothalamic–pituitary–ovarian axis through FSH and LH hormone secretion dysregulation caused by the hypothalamus and pituitary. PCOS has been linked to increased LH pulse frequency and amplitude, as well as ovarian defects caused by an internal issue that results in androgen overproduction. This does not, however, account for the fact that many women with PCOS do not have elevated LH levels (Dapas et al., 2020). Another theory about PCOS is that it results from a recurring ovarian failure cycle. In the event of no ovulation, the ovary releases more testosterone to compensate for the lack of progesterone, which, in turn, implies no ovulation (Wang et al., 2022).

From an evolutionary perspective, PCOS results from a genetic mismatch between previously neutral variations that developed in physically demanding lifestyle contexts and may become deleterious in sedentary, industrialized environments. Therefore, the high frequency and severity of PCOS are associated with current sedentary and obesogenic settings (Charifson and Trumble, 2019). Women’s relative infertility favors mother and child survival by lengthening the time between pregnancies and lowering the number of children, and insulin resistance lead to resistance to stressors such as scarcity of food, wounds, and epidemics by increasing the availability of glucose for the brain. In obesogenic environments, it increases the risk of cardiometabolic disorders (Charifson and Trumble, 2019).

### 2.1 PCOS subtypes

A systematic classification of PCOS may enable better diagnosis, treatment, and management of the condition based on each woman’s unique needs. Two of the three are suggested by the Rotterdam criteria: polycystic ovarian morphology, hyperandrogenism, and oligo- or anovulation. This results in four unique phenotypes: 1) complete, i.e., all three requirements are met; 2) classic, i.e., anovulation and hyperandrogenism are present; 3) ovulatory, characterized by polycystic ovarian shape and hyperandrogenism; and 4) non-androgenic, defined by the existence of polycystic ovarian morphology and anovulation (Broekmans et al. 2006). A recent study distinguished between two primary PCOS subgroups using hormonal and metabolic markers. The reproductive subtype was identified by comparatively low body mass index (BMI) and insulin levels, as well as greater levels of LH and sex hormone-binding globulin (SHBG) and more severe infertility and irregular menstruation. In addition to having lower levels of SHBG and LH and higher levels of BMI, glucose, and insulin, the metabolic subtype was also linked to an increased risk of androgen excess symptoms, including hirsutism, acne, and hair loss. Significantly, distinct genetic connections were found for these subgroups, indicating potential differences in their origins. Variants in ovarian function-related genes, including PRDM2, IQCA1, BMPR1B, and CDH10, were linked to the reproductive subtype. Variants in glucose metabolism-related genes, including KCNH7 and FIGN, were linked to the metabolic subtype (Dapas et al., 2020). Thus, the primary cause of anovulation in obese PCOS women is likely hyperinsulinemia, whereas increased blood LH concentrations are more likely to produce anovulation in lean PCOS women (Barber, 2022).

## 3 PCOS and obesity

The percentage of overweight or obese women with PCOS ranges from 38% to 88%. Importantly, even a minor weight loss of 5% of body weight can improve a woman’s ovulatory function and symptoms of hyperandrogenism, as well as the reproductive and metabolic aspects of PCOS (Barber, 2022). Consequently, it is indisputable that obesity plays a key role in the development and maintenance of PCOS and that it significantly affects the degree of endocrine and clinical aspects of the illness in a large proportion of affected women. Obesity is independently associated with infertility and probably plays a part in the features of hyperandrogenism, even in women whose ovaries are normal (Barber et al., 2006). A meta-analysis of forty studies concluded that women with PCOS have a three-fold prevalence of type 2 diabetes mellitus and impaired glucose tolerance (Kakoly et al., 2018). Numerous PCOS symptoms, such as insulin resistance, elevated blood glucose, and reproductive issues., can be made worse by obesity. Although the exact mechanism behind the association between obesity and PCOS is uncertain, one likely mechanism is selective insulin resistance (Barber, 2022). PCOS increases the chance of getting diabetes, gestational diabetes, heart disease, high blood pressure, high cholesterol, and sleep apnea. Being obese can make a person considerably more susceptible to acquiring all of these conditions. Obesity and PCOS have been shown to share genetic similarities, including variations in the FTO gene locus. A Mendelian randomization study utilizing genetic loci found weak evidence for obesity causal for PCOS, but not the other way around (Brower et al., 2019).

### 3.1 Hormonal imbalance and metabolic syndrome

Comparing PCOS women to BMI-matched controls, more accumulation of abdominal fat is observed. Nonetheless, imaging studies have revealed that the distribution of fat was comparable in PCOS and control groups, indicating that central obesity might exist independently of PCOS (Zhu et al., 2021). Visceral fat has high metabolic activity and secretes a variety of hormones and cytokines that can disrupt insulin signaling, induce inflammation, and result in a chronic low-grade inflammatory and insulin-resistant state (Zhao et al., 2023). Adipose tissue secretes leptin, which acts on the hypothalamus to inhibit appetite and increase energy expenditure. Higher amounts of leptin in the bloodstream in obese women may cause a long-term downregulation of LEPR in the brain. Research conducted *in vitro* has demonstrated that leptin impacts steroidogenic pathways in granulosa cells and reduces the synthesis of progesterone and estrogen in a dose-dependent manner (Brannian et al., 1999). A total of 65%–95% of women with PCOS, including the majority of overweight and obese women and more than half of women of normal weight, have compensatory hyperinsulinemia and insulin resistance. It indicates that insulin resistance in PCOS is tissue-specific rather than a general phenomenon. While the ovary, adrenal glands, and liver continue to be insulin-sensitive, skeletal muscle and adipose tissue develop insulin resistance, leading to decreased glucose absorption and increased lipolysis, respectively. Hyperinsulinemia is a compensatory reaction to insulin resistance common in PCOS. The ovaries and adrenal glands are stimulated indirectly by hyperinsulinemia, which leads to an increase in androgen production. More precisely, when LH is stimulated, excess insulin increases the production of androgen in the ovarian theca cells, leading to follicular arrest and, thereby, anovulation. Nevertheless, not all obese women with oligomenorrhea exhibit hyperandrogenism, but they have increased LH pulse frequency, similar to PCOS women (Yoo et al., 2006). SHBG secreted by the liver, the main protein that binds testosterone in the serum, is suppressed by hyperinsulinemia (Silvestris et al., 2018). The National Institute for Health and Care Excellence states that since there is a substantial inverse relationship between SHBG levels and fasting serum insulin in obese women, measuring SHBG levels can serve as a proxy measure for the severity of hyperinsulinemia in PCOS-affected women (Daka et al., 2013). Consequently, following weight loss, there was a substantial negative correlation between SHBG concentrations and insulin levels. Remarkably, insulin did not suppress the SHBG level in cases of extreme insulin resistance (Akin et al., 2007).

## 4 Comorbidities with PCOS

PCOS highlights a multi-systemic defect involving gonadal, metabolic, and neuroendocrine components. The most prevalent comorbidities associated with PCOS include long-term metabolic risks and emotional well-being [(Table 1, Kurki et al. (2023)]. PCOS comorbidities are not systematically considered in clinical care. Cardiometabolic disorders, including type 2 diabetes, hypertension, dyslipidemia, and cardiovascular disease, are the most prevalent comorbidities. Below is a brief description of other common comorbidities linked to PCOS.

**TABLE 1:** Comorbidities associated with PCOS. Data are obtained from the FinnGen population study (Kurki et al., 2023).

PCOS-associated comorbidity	Hazard ratio	*p*-value
Hirsutism	50.87	2.3 e–50
Female infertility, associated with anovulation	50.08	≤ 1 e–100
Hypertrichosis	44.52	3.9 e–47
Amenorrhea	29.88	≤ 1 e–100
Non-inflammatory disorders of the female genital tract	27.72	≤ 1 e–100
Oligomenorrhea	20.66	4.3 e–38
Female infertility	19.49	≤ 1 e–100
Medical treatment for female infertility	13.80	1.1 e–61
Female infertility, cervical, vaginal, other, or unspecified origin	13.56	6.0e–90
Type 2 diabetes, definitions combined, including Avohilmo	8.60	1.4 e–38
Obesity due to excess calories	8.54	4.2 e–32
Type 2 diabetes, strict (excluding DM1)	8.50	4.3 e–38
Type 2 diabetes, definitions combined	8.49	4.5 e–38
Obesity	7.41	6.7 e–35
Obesity and other hyperalimentation	7.38	8.6 e–35
Other nutritional deficiencies	7.17	3.8 e–33
Type 2 diabetes	6.87	2.4 e–36
Endometriosis diagnosis and infertility	5.52	1.4 e–9
Diabetes, varying definitions	4.76	8.6 e–38
Obesity, other/unspecified	4.59	2.9 e–9
Diabetes mellitus	4.36	7.8 e–33
Excessive, frequent, and irregular menstruation	4.32	1.0 e–20
Pain (limb, back, neck, head, and abdominal)	4.25	1.6 e–45
Abdominal and pelvic pain	3.88	1.5 e–39
Migraine with aura	3.86	1.7 e–39
Symptoms and signs involving the digestive system and abdomen	3.83	2.0 e–39
Persons encountering health services related to reproduction	3.82	7.3 e–27
Gestational diabetes (for exclusion)	3.66	9.8 e–11
Disorders of the thyroid gland	3.55	2.9 e–18
Disorders of the skin appendages	3.46	1.2 e–10
Hypothyroidism, other/unspecified	3.44	1.4 e–15
Migraine without aura	3.43	6.5 e–7
Hypothyroidism (congenital or acquired)	3.43	1.7 e–15
Migraine	3.35	5.0 e–12
Diabetes mellitus in pregnancy	3.35	1.8 e–9
Other specified and unspecified personality disorders	3.33	1.7 e–4
Bipolar affective disorders	3.21	1.4 e–6
*Postpartum* depression	3.20	3.3 e–9
Eating disorders	3.20	9.7 e–6
Other abnormal products of conception	3.18	2.1 e–9
Hypothyroidism, strict autoimmune	3.18	3.2 e–12
Depression	3.18	9.0 e–21
Phobic anxiety disorders	3.14	9.8 e–5
Endometriosis of the ovary	3.13	2.0 e–4
Mood disorders	3.13	1.3 e–21
Mood-affective disorders	3.13	1.3 e–21
Other maternal disorders predominantly related to pregnancy	3.13	3.5 e–13
Type 1 diabetes with ophthalmic complications	3.11	1.7 e–5
Ovarian cyst	3.04	1.2 e–8

### 4.1 Psycho-social disorders

PCOS symptoms can have a significant influence on a person’s life and may affect their mood or general well-being. Accordingly, it is associated with a higher risk of several mental health disorders (Table 1), including depression, anxiety, eating disorders, low self-esteem, and negative body image. In women with PCOS, hirsutism, i.e., excess terminal hair development in male-typical body areas, had the greatest effect on the quality of life metrics relating to health (Khomami et al., 2015).

### 4.2 Endometrial cancer

Endometrial carcinoma is the sixth most common cancer in women, and because of their higher frequency of obesity and extended oligomenorrhea or amenorrhea, women with PCOS appear to have a three- to four-fold increased risk of endometrial cancer. Thinning or irregular menstruation can lead to endometrial accumulation and thickening. In fact, a prognostic signature for endometrial cancer was shown to be derived from genes involved in the production of steroid hormones (Zhang et al., 2023).

### 4.3 Non-alcoholic fatty liver disease

Non-alcoholic fatty liver disease (NAFLD) is more common in PCOS patients (odds ratio 2.54). Even after controlling for confounding variables, the prevalence of NAFLD is greater in PCOS women with hyperandrogenism (classic phenotype) than in women with PCOS without hyperandrogenism. In women with PCOS, serum androgens were independent predictors of NAFLD (Rocha et al., 2017). Both PCOS and NAFLD have enhanced pathways linked to immunity and inflammation (Chen et al., 2022). A different study that used Biobank data discovered that there was less evidence linking genetically predicted NAFLD to a higher risk of PCOS development. The relationship between NAFLD and PCOS may be mediated by sex hormones and fasting insulin (Liu et al., 2023).

### 4.4 Sleep apnea

A systematic review and meta-analysis showed a clear link between PCOS and obstructive sleep apnea, particularly in obese individuals (Vgontzas et al., 2001). By increasing upper airway collapsibility and/or reducing the sensitivity and responsiveness of the ventilatory chemo-receptors, hyperandrogenism, and low progesterone levels may contribute to the pathophysiology of obstructive sleep apnea. It is unclear, nevertheless, how PCOS and obesity interact, leading to sleep apnea (Kahal et al., 2017).

## 5 Discussion

Currently, PCOS is typically diagnosed following the appearance of one or more of the three primary symptoms, namely, oligo- or anovulation, hyperandrogenism, and polycystic ovarian morphology, and the clinical diagnosis is essentially an exclusion diagnosis. There is a wider recognition now that PCOS is a multi-system disorder with neuroendocrine, gonadal, and metabolic components and, therefore, likely emerging through multiple etiologies. One approach to creating evidence-based, shared, standardized standards for PCOS diagnosis and treatment is through the systematic stratification of the condition. The PCOS subtyping has led to the identification of two subtypes: obesity and reproductive (Dapas et al., 2020). It is important to note that many women do not fit either reproductive or obese phenotypes. PCOS and obesity are frequently coexisting conditions that may have a bidirectional (or even causal) relationship (Barber, 2022). Given that all obese women do not suffer from PCOS and *vice versa*, this suggests that PCOS might originate from a weak reproductive defect exacerbated by obesity or through major hormonal dysregulation. Phenotypic heterogeneity exists throughout the reproductive lifespan, further complicating the diagnosis by the overlap between PCOS symptoms and normal oscillations in a woman’s menstrual cycle and metabolism during the menarche and menopausal transition. In summary, systematic sub-classification of PCOS for understanding distinct PCOS etiologies would guide evidence-based precision diagnosis and treatment for PCOS.

There is strong epidemiological evidence for the association of various disorders, especially metabolic syndrome and mental health disorders, with PCOS. There is, however, a lack of understanding as to whether these pathologies manifest only in specific PCOS subtypes. There are ethnic differences in impaired glucose tolerance among PCOS women (Kakoly et al., 2018). Racial and ethnic differences (often contradictory) are noted across nearly all aspects of PCOS discussed above, specifically metabolic dysfunction and associated psycho-social disorders (VanHise et al., 2023). Large omics-based studies of age and ethnic diversity in women, together with clinical and epidemiological data, will allow the identification of clinically relevant PCOS subtypes, together with the comorbidities associated with these subtypes. The development of subtype-specific and potentially age- and ethnicity-specific diagnostic and treatment options will be crucial toward evidence-based guidelines for early screening for an accurate diagnosis and managing PCOS throughout life. This strategy should minimize unnecessary testing and maximize healthcare resources. The individual requirements and preferences of women, combined with the most recent clinical expertise to provide tailored therapeutic care, will enable them to manage their own health in the best possible way.

## References

[B1] Akin F. Bastemir M. Kaptanoglu B. (2007). Relationship between insulin and sex hormone-binding globulin levels during weight loss in obese women. Ann. Nutr. Metabolism 51, 557–562. 10.1159/000114210 18227624

[B2] Azziz R. Carmina E. Chen Z. Dunaif A. Laven J. S. E. Legro R. S. (2016). Polycystic ovary syndrome. Nat. Rev. Dis. Prim. 2, 16057. 10.1038/nrdp.2016.57 27510637

[B3] Azziz R. Carmina E. Dewailly D. Diamanti-Kandarakis E. Escobar-Morreale H. F. Futterweit W. (2006). Positions statement: criteria for defining polycystic ovary syndrome as a predominantly hyperandrogenic syndrome: an Androgen Excess Society guideline. J. Clin. Endocrinol. Metabolism 91, 4237–4245. 10.1210/jc.2006-0178 16940456

[B4] Balen A. H. Laven J. S. E. Tan S.-L. Dewailly D. (2003). Ultrasound assessment of the polycystic ovary: international consensus definitions. Hum. Reprod. Update 9, 505–514. 10.1093/humupd/dmg044 14714587

[B5] Barber T. M. (2022). Why are women with polycystic ovary syndrome obese? Br. Med. Bull. 143, 4–15. 10.1093/bmb/ldac007 35284917 PMC9494255

[B6] Barber T. M. McCarthy M. I. Wass J. A. H. Franks S. (2006). Obesity and polycystic ovary syndrome. Clin. Endocrinol. 65, 137–145. 10.1111/j.1365-2265.2006.02587.x 16886951

[B7] Brannian J. D. Zhao Y. McElroy M. (1999). Leptin inhibits gonadotrophin-stimulated granulosa cell progesterone production by antagonizing insulin action. Hum. Reprod. 14, 1445–1448. 10.1093/humrep/14.6.1445 10357956

[B8] Broekmans F. J. Knauff E. a. H. Valkenburg O. Laven J. S. Eijkemans M. J. Fauser B. C. J. M. (2006). PCOS according to the Rotterdam consensus criteria: change in prevalence among WHO-II anovulation and association with metabolic factors. BJOG Int. J. obstetrics Gynaecol. 113, 1210–1217. 10.1111/j.1471-0528.2006.01008.x 16972863

[B9] Brower M. A. Hai Y. Jones M. R. Guo X. Chen Y. D. I. Rotter J. I. (2019). Bidirectional Mendelian randomization to explore the causal relationships between body mass index and polycystic ovary syndrome. Hum. Reprod. 34, 127–136. 10.1093/humrep/dey343 30496407 PMC6295958

[B10] Butt M. S. Saleem J. Aiman S. Zakar R. Sadique I. Fischer F. (2022). Serum anti-Müllerian hormone as a predictor of polycystic ovarian syndrome among women of reproductive age. BMC Women’s Health 22, 199. 10.1186/s12905-022-01782-2 35643521 PMC9148456

[B11] Charifson M. A. Trumble B. C. (2019). Evolutionary origins of polycystic ovary syndrome: an environmental mismatch disorder. Evol. Med. Public Health 2019, 50–63. 10.1093/emph/eoz011 31367382 PMC6658700

[B12] Chen Y. Ma L. Ge Z. Pan Y. Xie L. (2022). Key genes associated with non-alcoholic fatty liver disease and polycystic ovary syndrome. Front. Mol. Biosci. 9, 888194. 10.3389/fmolb.2022.888194 35693550 PMC9174783

[B13] Cowan S. Lim S. Alycia C. Pirotta S. Thomson R. Gibson-Helm M. (2023). Lifestyle management in polycystic ovary syndrome – beyond diet and physical activity. BMC Endocr. Disord. 23, 14. 10.1186/s12902-022-01208-y 36647089 PMC9841505

[B14] Daka B. Rosen T. Jansson P. A. Råstam L. Larsson C. A. Lindblad U. (2013). Inverse association between serum insulin and sex hormone-binding globulin in a population survey in Sweden. Endocr. Connect. 2, 18–22. 10.1530/EC-12-0057 23781314 PMC3680959

[B15] Danaei Mehr H. Polat H. (2022). Diagnosis of polycystic ovary syndrome through different machine learning and feature selection techniques. Health Technol. 12, 137–150. 10.1007/s12553-021-00613-y

[B16] Dapas M. Lin F. T. J. Nadkarni G. N. Sisk R. Legro R. S. Urbanek M. (2020). Distinct subtypes of polycystic ovary syndrome with novel genetic associations: an unsupervised, phenotypic clustering analysis. PLOS Med. 17, e1003132. 10.1371/journal.pmed.1003132 32574161 PMC7310679

[B17] Day F. Karaderi T. Jones M. R. Meun C. He C. Drong A. (2018). Large-scale genome-wide meta-analysis of polycystic ovary syndrome suggests shared genetic architecture for different diagnosis criteria. PLOS Genet. 14, e1007813. 10.1371/journal.pgen.1007813 30566500 PMC6300389

[B18] Dumont A. Robin G. Catteau-Jonard S. Dewailly D. (2015). Role of anti-müllerian hormone in pathophysiology, diagnosis and treatment of polycystic ovary syndrome: a review. Reproductive Biol. Endocrinol. 13, 137. 10.1186/s12958-015-0134-9 PMC4687350 26691645

[B19] Escobar-Morreale H. F. Luque-Ramírez M. San Millán J. L. (2005). The molecular-genetic basis of functional hyperandrogenism and the polycystic ovary syndrome. Endocr. Rev. 26, 251–282. 10.1210/er.2004-0004 15561799

[B20] Fauser B. C. J. M. Tarlatzis B. C. Rebar R. W. Legro R. S. Balen A. H. (2012). Consensus on women’s health aspects of polycystic ovary syndrome (PCOS): the Amsterdam ESHRE/ASRM-Sponsored 3rd PCOS Consensus Workshop Group. Hum. Reprod. 27, 14–24. 10.1093/humrep/der396 22147920

[B21] Hoeger K. M. Dokras A. Piltonen T. (2021). Update on PCOS: consequences, challenges, and guiding treatment. J. Clin. Endocrinol. Metabolism 106, e1071–e1083. 10.1210/clinem/dgaa839 33211867

[B22] Kahal H. Kyrou I. Tahrani A. A. Randeva H. S. (2017). Obstructive sleep apnoea and polycystic ovary syndrome: a comprehensive review of clinical interactions and underlying pathophysiology. Clin. Endocrinol. 87, 313–319. 10.1111/cen.13392 28640938

[B23] Kakoly N. S. Khomami M. B. Joham A. E. Cooray S. D. Misso M. L. Norman R. J. (2018). Ethnicity, obesity and the prevalence of impaired glucose tolerance and type 2 diabetes in PCOS: a systematic review and meta-regression. Hum. Reprod. Update 24, 455–467. 10.1093/humupd/dmy007 29590375

[B24] Karimzadeh M. A. Javedani M. (2010). An assessment of lifestyle modification versus medical treatment with clomiphene citrate, metformin, and clomiphene citrate–metformin in patients with polycystic ovary syndrome. Fertil. Steril. 94, 216–220. 10.1016/j.fertnstert.2009.02.078 19463994

[B25] Khomami M. B. Tehrani F. R. Hashemi S. Farahmand M. Azizi F. (2015). Of PCOS symptoms, hirsutism has the most significant impact on the quality of life of Iranian women. PLOS ONE 10, e0123608. 10.1371/journal.pone.0123608 25874409 PMC4398498

[B26] Kiconco S. Earnest A. Enticott J. Hart R. Mori T. A. Hickey M. (2023). Normative cut-offs for polycystic ovary syndrome diagnostic features in adolescents using cluster analysis. Eur. J. Endocrinol. 188, 494–502. 10.1093/ejendo/lvad055 37243570

[B27] Kurki M. I. Karjalainen J. Palta P. Sipilä T. P. Kristiansson K. Donner K. M. (2023). FinnGen provides genetic insights from a well-phenotyped isolated population. Nature 613, 508–518. 10.1038/s41586-022-05473-8 36653562 PMC9849126

[B28] Liu D. Gao X. Pan X.-F. Zhou T. Zhu C. Li F. (2023). The hepato-ovarian axis: genetic evidence for a causal association between non-alcoholic fatty liver disease and polycystic ovary syndrome. BMC Med. 21, 62. 10.1186/s12916-023-02775-0 36800955 PMC9940436

[B29] McCartney C. R. Marshall J. C. (2016). CLINICAL PRACTICE. Polycystic ovary syndrome. N. Engl. J. Med. 375, 54–64. 10.1056/NEJMcp1514916 27406348 PMC5301909

[B30] Merkin S. S. Phy J. L. Sites C. K. Yang D. (2016). Environmental determinants of polycystic ovary syndrome. Fertil. Steril. 106, 16–24. 10.1016/j.fertnstert.2016.05.011 27240194

[B31] Riestenberg C. Jagasia A. Markovic D. Buyalos R. P. Azziz R. (2022). Health care-related economic burden of polycystic ovary syndrome in the United States: pregnancy-related and long-term health consequences. J. Clin. Endocrinol. Metabolism 107, 575–585. 10.1210/clinem/dgab613 34546364

[B32] Rocha A. L. L. Faria L. C. Guimarães T. C. M. Moreira G. V. Cândido A. L. Couto C. A. (2017). Non-alcoholic fatty liver disease in women with polycystic ovary syndrome: systematic review and meta-analysis. J. Endocrinol. Investigation 40, 1279–1288. 10.1007/s40618-017-0708-9 28612285

[B33] Rotterdam ESHRE/ASRM-Sponsored PCOS Consensus Workshop Group (2004). Revised 2003 consensus on diagnostic criteria and long-term health risks related to polycystic ovary syndrome. Fertil. Steril. 81, 19–25. 10.1016/j.fertnstert.2003.10.004 14711538

[B34] Silvestris E. De Pergola G. Rosania R. Loverro G. (2018). Obesity as disruptor of the female fertility. Reproductive Biol. Endocrinol. 16, 22. 10.1186/s12958-018-0336-z PMC5845358 29523133

[B35] Suha S. A. Islam M. N. (2022). An extended machine learning technique for polycystic ovary syndrome detection using ovary ultrasound image. Sci. Rep. 12, 17123. 10.1038/s41598-022-21724-0 36224353 PMC9556522

[B36] VanHise K. Wang E. T. Norris K. Azziz R. Pisarska M. D. Chan J. L. (2023). Racial and ethnic disparities in polycystic ovary syndrome. Fertil. Steril. 119, 348–354. 10.1016/j.fertnstert.2023.01.031 36702345 PMC11354608

[B37] Vgontzas A. N. Legro R. S. Bixler E. O. Grayev A. Kales A. Chrousos G. P. (2001). Polycystic ovary syndrome is associated with obstructive sleep apnea and daytime sleepiness: role of insulin resistance ^1^ . J. Clin. Endocrinol. Metabolism 86, 517–520. 10.1210/jcem.86.2.7185 11158002

[B38] Vink J. M. Sadrzadeh S. Lambalk C. B. Boomsma D. I. (2006). Heritability of polycystic ovary syndrome in a Dutch twin-family study. J. Clin. Endocrinol. Metabolism 91, 2100–2104. 10.1210/jc.2005-1494 16219714

[B39] Wang Y. Leung P. Li R. Wu Y. Huang H. (2022). Editorial: polycystic ovary syndrome (PCOS): mechanism and management. Front. Endocrinol. 13, 1030353. 10.3389/fendo.2022.1030353 PMC9585504 36277701

[B40] Yoo R. Y. Dewan A. Basu R. Newfield R. Gottschalk M. Chang R. J. (2006). Increased luteinizing hormone pulse frequency in obese oligomenorrheic girls with no evidence of hyperandrogenism. Fertil. Steril. 85, 1049–1056. 10.1016/j.fertnstert.2005.09.037 16580394

[B41] Zhang Y. Hu Y. Yu J. Xie X. Jiang F. Wu C. (2023). Landscape of PCOS co-expression gene and its role in predicting prognosis and assisting immunotherapy in endometrial cancer. J. Ovarian Res. 16, 129. 10.1186/s13048-023-01201-6 37393293 PMC10315044

[B42] Zhao H. Zhang J. Cheng X. Nie X. He B. (2023). Insulin resistance in polycystic ovary syndrome across various tissues: an updated review of pathogenesis, evaluation, and treatment. J. Ovarian Res. 16, 9. 10.1186/s13048-022-01091-0 36631836 PMC9832677

[B43] Zhu S. Li Z. Hu C. Sun F. Wang C. Yuan H. (2021). Imaging-based body fat distribution in polycystic ovary syndrome: a systematic review and meta-analysis. Front. Endocrinol. 12, 697223. 10.3389/fendo.2021.697223 PMC8458943 34566888

